# High-Intensity Interval Training in Normobaric Hypoxia Leads to Greater Body Fat Loss in Overweight/Obese Women than High-Intensity Interval Training in Normoxia

**DOI:** 10.3389/fphys.2018.00060

**Published:** 2018-02-07

**Authors:** Alba Camacho-Cardenosa, Marta Camacho-Cardenosa, Martin Burtscher, Ismael Martínez-Guardado, Rafael Timon, Javier Brazo-Sayavera, Guillermo Olcina

**Affiliations:** ^1^Faculty of Sport Sciences, University of Extremadura, Cáceres, Spain; ^2^Medical Section, Department of Sport Science, University of Innsbruck, Innsbruck, Austria; ^3^Instituto Superior de Educación Física, Universidad de la República, Montevideo, Uruguay

**Keywords:** normobaric hypoxia, body mass loss, obese, exercise, high-intensity

## Abstract

A moderate hypoxic stimulus is considered a promising therapeutic modality for several pathological states including obesity. There is scientific evidence suggesting that when hypoxia and physical activity are combined, they could provide benefits for the obese population. The aim of the present study was to investigate if exposure to hypoxia combined with two different protocols of high-intensity interval exercise in overweight/obese women was more effective compared with exercise in normoxia. Study participants included 82 overweight/obese women, who started a 12 week program of 36 sessions, and were randomly divided into four groups: (1) aerobic interval training in hypoxia (AitH; FiO_2_ = 17.2%; *n* = 13), (2) aerobic interval training in normoxia (AitN; *n* = 15), (3) sprint interval training in hypoxia (SitH; *n* = 15), and (4) sprint interval training in normoxia (SitN; *n* = 18). Body mass, body mass index, percentage of total fat mass, muscle mass, basal metabolic rate, fat, and carbohydrate oxidation, and fat and carbohydrate energy were assessed. Outcomes were measured at baseline (T1), after 18 training sessions (T2), 7 days after the last session (T3), and 4 weeks after the last session (T4). The fat mass in the SitH group was significantly reduced compared with the SitN group from T1 to T3 (*p* < 0.05) and from T1 to T4 (*p* < 0.05) and muscle mass increased significantly from T1 to T4 (*p* < 0.05). Fat mass in the AitH group decreased significantly (*p* < 0.01) and muscle mass increased (*p* = 0.022) compared with the AitN group from T1 to T4. All training groups showed a reduction in the percentage of fat mass, with a statistically significant reduction in the hypoxia groups (*p* < 0.05). Muscle mass increased significantly in the hypoxia groups (*p* < 0.05), especially at T4. While fat oxidation tended to increase and oxidation of carbohydrates tended to decrease in both hypoxia groups, the tendency was reversed in the normoxia groups. Thus, high-intensity interval training under normobaric intermittent hypoxia for 12 weeks in overweight/obese women seems to be promising for reducing body fat content with a concomitant increase in muscle mass.

## Introduction

According to the World Health Organization (WHO, 2006), overweight and obesity are defined as abnormal or excessive fat accumulation that may impair health. Although there is a global obesity pandemic, the prevalence of being overweight and obesity among men and women is different, and overall, more women are obese than men (Kanter and Caballero, 2012). Being overweight and obesity are major public health concerns as they are key risk factors for a number of chronic diseases (Malnick and Knobler, 2006); thus, effective fat loss strategies are required (Jakicic et al., 2001). In this sense, increasing the level of physical activity is likely a crucial intervention for efficient prevention and treatment of obesity (Girard et al., 2017). During the last few decades, it has been well-established that exercise is a good strategy for maintaining weight loss (Bouchard et al., 1993). However, current weight management exercise strategies are ineffective in the long-term because after 6 months they often produce a plateau in body mass or even a recovery of lost body mass (Urdampilleta et al., 2012). This outcome is facilitated by the fact that adherence to physical activity often declines over time (Urdampilleta et al., 2012). Countless studies have shown that lack of time, motivation, and adherence are the most commonly cited reasons for not exercising (Smith-Ryan et al., 2016). On other hand, obese patients would have to exercise relatively more than lean individuals to increase exercise intensity and comply with the World Health Organization (WHO) recommended guidelines (Girard et al., 2017). Thus, the increased mechanical demand during exercise for obese populations may be deleterious on lower limb joints and limit the functional capabilities compared to healthy and normal weight populations (Wearing et al., 2006). Altogether, these impediments provoke non-adherence by obese patients to current exercise recommendations.

In this context, moderate hypoxia is presently considered a favourable treatment option for some diseases such as obesity (Urdampilleta et al., 2012; Kayser and Verges, 2013; Millet et al., 2016; Camacho-Cardenosa et al., 2017). Recently, it was reported that hypoxia exposure may lead to considerable (3%) body mass loss and improve other health conditions, including some cardiorespiratory parameters (Netzer et al., 2008; Kayser and Verges, 2013; Kong et al., 2016), likely due to a decrease in food intake (hypoxia-induced appetite reduction) (Westerterp and Kayser, 2006) and increased energy expenditure (Kayser and Verges, 2013). A likely explanation for higher fat loss with this novel therapy is the enhanced lipid metabolism, which might be due to the intermittent hypoxic conditions (Wiesner et al., 2010; Workman and Basset, 2012). These changes may be modulated via hypoxia-inducible factor 1α (HIF-1α), which is not activated to the same extent by passive hypoxic exposure (during rest) or by active hypoxic exposure (during exercise) (Millet et al., 2016). There is scientific evidence suggesting that a lower degradation of HIF-1α results when hypoxia and physical activity are combined (Urdampilleta et al., 2012). It seems likely that the main underlying mechanism is the larger hypoxemia resulting from the combination of muscle deoxygenation (exercise) and systemic desaturation (hypoxia) (Millet et al., 2016). Recent findings show that the recovery time characteristics in intermittent hypoxia exercise programs may contribute to the rapid body mass loss (Kelly and Basset, 2017). In this sense, the amount of depleted glycogen is correlated with lipid oxidation post-exercise. Thus, exercise strategies that require a large amount of endogenous glucose may be an effective strategy to increase lipid oxidation post-exercise and improve body fat mass (Kelly and Basset, 2017). Trombold et al. (2013) showed that high intensity exercise (2 min at 25% and 2 min at 90% of VO_2_ max) was more effective than moderate intensity exercise (50% of VO_2_ max for 60 min) in increasing lipid oxidation post-exercise, which was related to the increased muscle glycogen consumption during the vigorous effort. High-intensity intermittent training (HIIT) refers to intermittent exercise that involves alternating brief (6 s to 4 min), high-intensity anaerobic exercise (85–250% VO_2_ max) separated by brief, but slightly longer bouts (10 s to 5 min) of low-intensity aerobic (20 to 40% VO_2_ max) rest (Batacan et al., 2017). Supramaximal interval training (SIT), which involves 30 s of “all out” supramaximal sprints, or aerobic interval training (AIT), which requires longer (3–4 min) lower intensity intervals (Smith-Ryan et al., 2015), are two distinct types of HIIT used to reduce cardiometabolic disease risk (Kessler et al., 2012). Further research in this area is required to validate these findings and differentiate the effect of hypoxia at different intensities (Hobbins et al., 2017). In addition to the possible benefits mentioned previously, a recent study reported that vigorous exercise could be effective for improving muscle mass, showing an additional benefit with respect to other studies that used moderate-intensity exercise (Oh et al., 2017). This could be especially important in obese patients where reduced muscle mass (sarcopenia) is common (Gallagher and DeLegge, 2011). Muscles have increased fat infiltration, which stimulates muscle catabolism by expression of proinflammatory cytokines rather than exercise-inducible myokines (Walsh, 2009).

On the other hand, the benefits result in HIF activation may be gender-dependent. In normoxic conditions, previous studies have demonstrated gender specific differences in the metabolic response to submaximal endurance exercise: higher fat utilisation during endurance exercises was apparent in females compared with males (Carter et al., 2001). In hypoxic conditions, previous studies have suggested that oestrogen could downregulate HIF activity. HIF may induce a shift in metabolism in favour of glucose utilisation, which is higher in men compared with women, who exhibit a greater reliance on fatty acids for metabolism (Palmer and Clegg, 2014). Thus, it should be considered that gender may affect the results of this type of exercise strategy.

Given that current exercise strategies do not meet the needs of the overweight/obese, clinically validated and specific alternatives are required. Based on the above, we reasoned that the combination of hypoxia and high-intensity exercise may result in synergistic effects on body composition and metabolism and thus, would be an appropriate and time-metabolic effective form of exercise training for overweight/obese women. Thus, the aim of the present study was to investigate if exposure to hypoxia combined with two different protocols of high-intensity interval exercise in overweigh/obese women would be more effective when compared with exercising in normoxia. We hypothesised that the addition of a hypoxic stimulus to high-intensity exercise would be more effective in reducing body fat than the same protocol performed in normoxic conditions.

## Materials and methods

### Participants

Participants were recruited through advertising in the community and the researchers' host institution, and personal contact. Inclusion criteria were assessed during a screening visit. Inclusion criteria were: BMI (body mass index) >25 kg/m^2^ (overweight or obese for adults according to WHO) or percentage fat mass (% fat) >29.9% [elevated risk of cardiovascular disease (ACSM, 2014)], absence of other associated diseases, pre-menopausal, sedentary (<2 bouts of exercise lasting a minimum of 30 min per bout per week), and have not been above 1,500 m during the last 3 months. The exclusion criteria were diseases not compatible with study exercise: myocardial infarction or stoke within 6 months before the start of the study, unstable angina pectoris, malignant hypertension, or chronic kidney disease.

**Figure 1:**
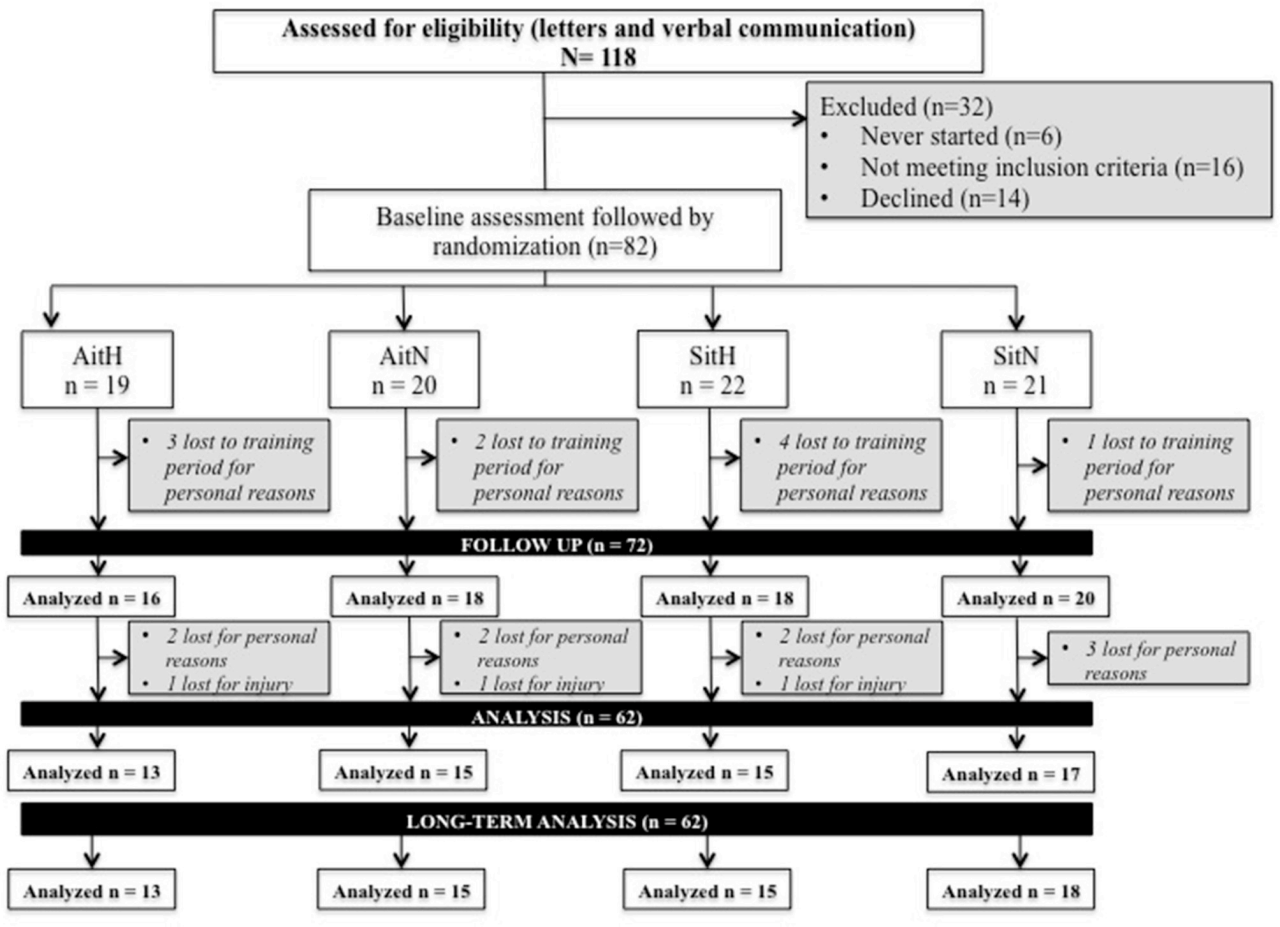
Flow of participants through each stage of the trial. AitH: Aerobic interval training Hypoxia group; AitN: Aerobic interval training Normoxia group; SitH: Sprint interval training Hypoxia group; SitN: Sprint interval training Normoxia group.

In total, 112 female volunteers were informed about the study procedures and, after verifying inclusion and exclusion criteria through an exam, the volunteers were asked to sign a declaration that they voluntarily consented to participate in this research. The eligible volunteers (*n* = 82; body mass: 78.12 ± 14.70 kg; BMI: 28.98 ± 5.19; % fat: 38.63 ± 5.78) started interval training treatment under normoxic or hypoxic conditions. Due to health problems (not related to the study protocol), occupational factors and other personal problems, 23 participants dropped out during the intervention period (see Figure 1).

The study was performed in line with the ethical standards of the Declaration of Helsinki. The Bioethical and Biosecurity Commission of the University of Extremadura approved the study protocol.

### Procedures

The study was designed as a randomised double-blind control study. There were separate intervention and assessment teams. The volunteers were randomly divided into four groups: (1) aerobic interval training in hypoxia (AitH; *n* = 13), in which aerobic interval training under normobaric hypoxic conditions was performed, (2) aerobic interval training in normoxia (AitN; *n* = 15) in which aerobic interval training under normoxic conditions was performed, (3) supramaximal interval training in hypoxia (SitH; *n* = 15) in which sprint interval training under normobaric hypoxic conditions was performed, and (4) supramaximal interval training in normoxia (SitN; *n* = 18) in which sprint interval training under normoxic conditions was performed. For 12 weeks, the volunteers completed an intervention supervised by an experienced member of the research group. Approximately 2 weeks prior to baseline measurements, participants reported to the laboratory for familiarisation with experimental trials and fitness and psychological testing. A general questionnaire was completed to collect medical and personal data before entering the study. All participants were assessed at four time points by a group of researchers who were blinded to the treatment assigned. Outcomes were evaluated at baseline (T1), after 18 training sessions (T2), in the 7 days after the last session (T3), and 4 weeks after the last session (T4). All time points for evaluations consisted of the same measurements.

### Exercise

All participants started the training protocol 1 week following baseline. During the 20-week study period, 36 sessions were completed within 12 weeks, 3 days per week. Sessions were scheduled with at least 1 day of rest between for optimal recovery (Mondays, Wednesdays, and Fridays) and participants were requested to train at the same time throughout the 36 sessions. At each session, an exercise physiologist recorded adherence, exercise workloads, and physiological responses in a daily training log. SpO_2_ was controlled regularly via pulse-oximeter (Konica Minolta, Japan). Heart rate (HR) was monitored during each session with a HR monitor (Team System, Polar Electro Oy). Participants rated their perceived exertion (0–10 scale) at the end of each training session (Borg, 1982).

#### HIIT

Participants performed two different HIIT protocols 3 days per weeks on a cycle ergometer (Ergoselect series 100/200, Ergoline GmbH, Bitz, Germany). The two exercise protocols were designed to maintain a progressive overall work over the 12 weeks of study. During the 1st and 2nd weeks, subjects finished three high-intensity intervals, four intervals between the 3rd and 5th weeks, five intervals between the 6th and 8th weeks, and six intervals during the last 9th and 12th weeks.

**Figure 2:**
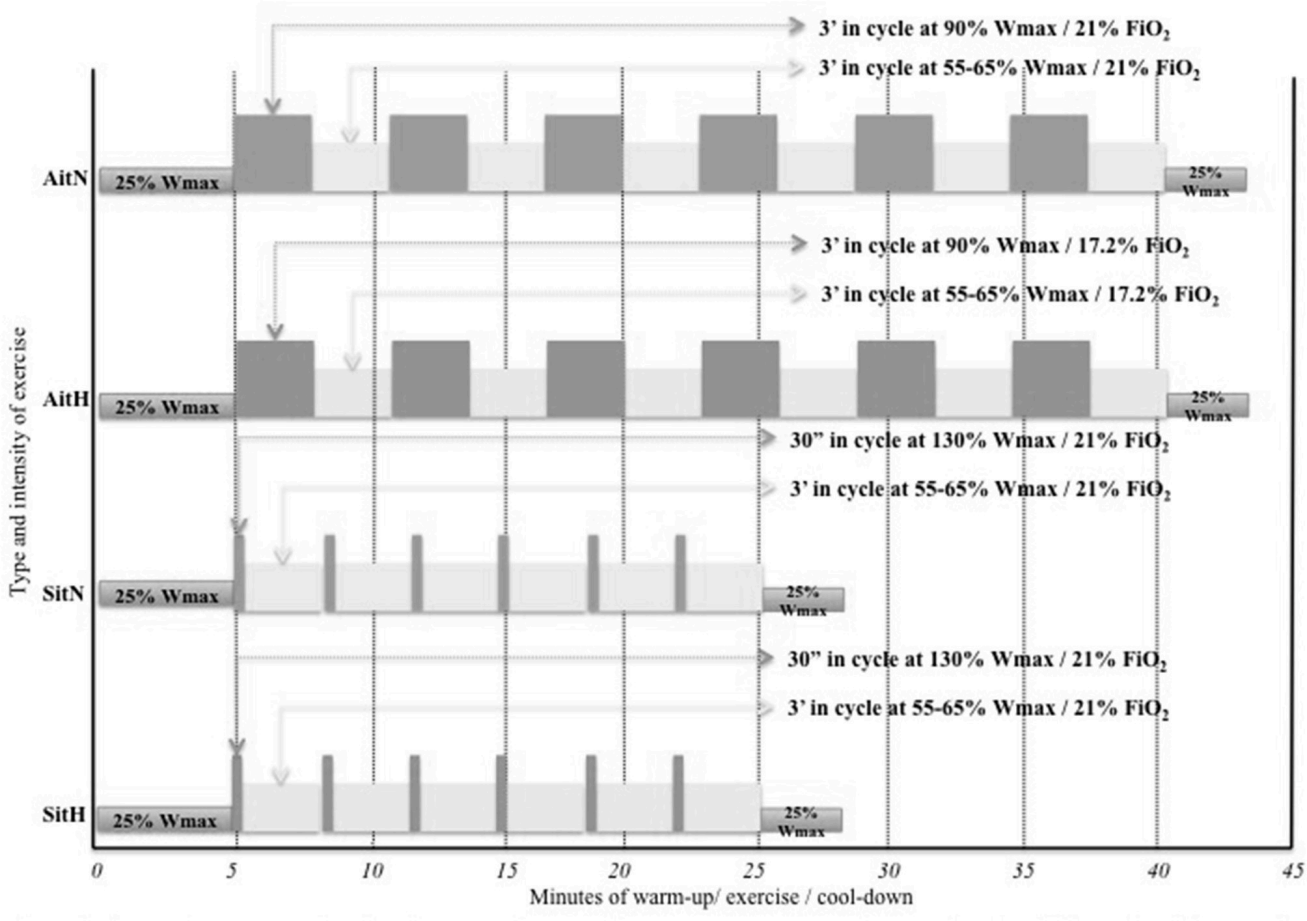
Schematic representation of the four exercise prescriptions allocated at randomisation. AitH: Aerobic interval training Hypoxia group; AitN: Aerobic interval training Normoxia group; SitH: Sprint interval training Hypoxia group; SitN: Sprint interval training Normoxia group; Wmax: maximal work-load; FiO_2_: inspired fraction of oxygen.

A 10 min warm-up and 3 min cool-down at 25% maximal workload (Wmax) were completed by the participants (Heydari et al., 2012; Keating et al., 2014; Lanzi et al., 2015). In the exercise protocol, the AitH and AitN groups performed 3 min of high intensity exercise [90% Wmax followed by 3 min of active recovery (55–65% Wmax)]. The SitH and SitN groups underwent 30 s of all-out (130% Wmax) followed by 3 min of active recovery at 55–65% Wmax (Wood et al., 2015). Maximal average exercise time per session was 41.5 min in the Ait groups and 29.62 min in the Sit groups. The exercise prescriptions are summarised in Figure 2.

To assess Wmax, all participants performed a maximal ramp incremental test to exhaustion (FATmax) (Lanzi et al., 2014, 2015) on the same cycle ergometer (Ergoselect series 100/200, Ergoline GmbH, Bitz, Germany) as the training.

#### Hypoxic stimulus

All training sessions were performed in two normobaric hypoxia chambers (CAT 310, Lousiville, Colorado, USA) built in the laboratory (24°C and 40% relative humidity). The AitH and SitH groups exercised at an inspired fraction of oxygen (FiO_2_) of 17.2 ± 0.3% corresponding to approximately 2,500 m while the AitN and SitN groups exercised at FiO_2_ of 20.9% corresponding to sea level in the laboratory. Oxygen content within the chamber could be reduced by insufflating nitrogen, which was produced from chamber air through a molecular sieve. FiO_2_ was controlled regularly with an electronic device (HANDI+, Maxtec, Salt Lake City, Utah, USA). Blinding of the subjects was done by covering any displays in the hypoxic rooms and running the ventilation system at the same power with closed windows. The blinding success was assessed by interviewing the subjects after the intervention.

### Outcome measures

In the four time points, assessments took place in one session. Participants attended the research laboratory after a minimum 8-h overnight fast, for basal metabolic rate (BMR), and anthropometric measurements.

#### Medical history and lifestyle questionnaires

Subjects in both groups were instructed to maintain their usual eating habits and physical activities during the study period. On their first and last visit to the laboratory, subjects provided the International Physical Activity Questionnaire (IPAQ) (Craig et al., 2003) and a 7-day diet inventory, which was analysed using the diet software Nutriber (Nutriber v1.1.1.r5, Funiber, Barcelona, Spain).

#### Basal metabolic rate

Following a minimum 8 h overnight fast, participants visited the laboratory in the morning and rested. Participants were also required to avoid strenuous activity for the previous 48 h. First, subjects rested quietly in a comfortable position in a quiet neutral environment. The mixing chamber pump was turned on and the plastic canopy was placed over the reclined subject's head and neck. One minute of data were allowed to expire before initiating formal data collection to allow for acclimation to the gas analyser (Metalyzer 3b, CORTEX Biophysik GmbH, Leipzig, Germany). Appropriate calibrations of the O_2_ and CO_2_ sensors and the volume transducer were performed before each measurement. The metabolic rate was truncated by 10-min out of 20-min data collection. The procedure discarded the first and last 5 min to nullify any metabolic rate fluctuation due to familiarisation with the facemask and the expected termination of data collection. Data points were collected every 5 s and steady-state was defined as the 10 min during which the volume of oxygen consumed, ventilation (VE), and respiratory quotient (RQ) did not vary 0.10% (Horner et al., 2001). BMR was calculated using Weir equations (Haugen et al., 2007):

$$\begin{array}{lll} \text{BMR}\left(\text{kcal}/\text{day}\right) & = & \left(3.9\times \text{V}{\text{O}}_{2}\left(\text{L}/\text{min}\right)\right)+\left(1.1\times \text{C}{\text{O}}_{2}\left(\text{L}/\text{min}\right)\right)\\ \;\times 1440. \end{array}$$

Fat oxidation (FAToxi), carbohydrate oxidation (CHOoxi), energy fat (FATene), and energy carbohydrate (CHOene) were calculated using stoichiometric equations (Weir, 1949) and appropriate energy equivalents, with the assumption that the urinary nitrogen excretion rate will be negligible (Brandou et al., 2005):

$$\begin{array}{l} \text{FAToxi}\left(\text{g}/\text{min}\right)=\left[\left(1.67\times \text{VC }{\text{O}}_{2}\right)-\left(1.67\times \text{VC C}{\text{O}}_{2}\right)\right]\\ \text{CHOoxi}\left(\text{g}/\text{min}\right)=\left[\left(4.55\times \text{VC C}{\text{O}}_{2}\right)-\left(3.21\times \text{VC }{\text{O}}_{2}\right)\right]\\ \text{FATene}=\left[\left(1.67\times \text{VC }{\text{O}}_{2}\right)-\left(1.67\times \text{VC C}{\text{O}}_{2}\right)\right]\times 9\\ \text{CHOene}=\left[\left(4.55\times \text{VC C}{\text{O}}_{2}\right)-\left(3.21\times \text{VC }{\text{O}}_{2}\right)\right]\times 4 \end{array}$$

#### Anthropometric measures and body composition

Anthropometric data comprised body mass and height measures to the nearest 0.1 kg and 0.5 cm, respectively. Body composition was determined by bioelectrical impedance analysis using a standardised body composition analyser (Tanita BC 418 MA, Tanita Corp., Tokyo, Japan) and included estimation of body fat percentage (% fat), muscle mass, and BMI (coefficient variation = 1.1% for body fat). Subjects maintained a standing position, with feet side-by-side on the scale and were barefoot. They wore sport clothes without metal objects.

#### Maximal exercise test

A maximal incremental test (FATmax) on a cycle ergometer (Ergoselect series 100/200, Ergoline GmbH) with gas analyser (Metalyzer 3b, CORTEX Biophysik GmbH) was performed. After a 5-min warm-up at 50 W and 1 min of rest were allowed to expire before initiating formal data collection to allow for acclimation to the apparatus, the test was initiated. Subjects started cycling at 30 W and the work rate was increased 15 W every 3 min until exhaustion. HR was recorded continuously during the test using a cardio belt (Polar H7 HR, Polar Electro Oy, Kempele, Finland) integrated with the gas analyser software (MetaSoft Studio, CORTEX systems, Leipzig, Germany). The measurement finished when at least three of the following four criteria were met (Wood et al., 2010): (1) a plateauing of VO_2_ (defined as an increase of no more than 2 mL·kg^−1^·min^−1^ with an increase in workload) during the latter stages of the exercise test, (2) an HR <90% of the age predicted maximum (220–age), (3) a RER >1.1, and (4) an inability to maintain the minimal required pedalling frequency (i.e., 60 rpm) despite maximum effort and verbal encouragement. Thus, the peak power was defined as maximal power achieved during the last 3-min step completed during the incremental test.

### Statistical analysis

Statistical analyses were performed using the statistical package SPSS v.20 for MAC (IBM, New York, USA). Data are presented as the mean ± standard deviation (SD). Standard statistical methods were used for the calculation of the mean and SDs. Kolmogorov–Smirnov tests were conducted to show the distribution of the studied variables and Levene's test was used for homogeneity of variance. Treatment effects were obtained for every variable as absolute change (T2/T3/T4 mean minus T1 mean). All data were normally distributed; a two-way repeated measures analysis of variance (ANOVA) was performed accounting for time and the interaction term between group and time. If a global difference over time appeared, a Bonferroni *post-hoc* analysis was used to identify where changes occurred. The *p* < 0.05 criterion was used for establishing statistical significance.

## Results

Demographic and physiological baseline characteristics for both treatment groups are provided in Table 1. No initial difference in body composition parameters was found via ANOVA. Because of the neutral training place, subjects could not discriminate if they were in hypoxia or normoxia. Blinding was successful as more than 60% of subjects did not guess their conditions correctly. There were no serious health problems reported by the subjects.

**Table 1:** Baseline characteristics of the sample.

	**AitN** **(*n* = 13)**	**AitH** **(*n* = 13)**	**SitN** **(*n* = 15)**	**SitH** **(*n* = 18)**	***P*-value**
	**Mean ± SD**	**Mean ± SD**	**Mean ± SD**	**Mean ± SD**	
Age, years	43.14 ± 7.67	44.43 ± 7.18^b^	40.05 ± 8.66	37.40 ± 10.25^a^	**0.038**
Height, m	1.64 ± 0.06	1.63 ± 0.06	1.65 ± 0.07	1.63 ± 0.05	NS
Body mass, kg	80.41 ± 16.27	80.10 ± 18.88	77.94 ± 11.31	73.73 ± 11.11	NS
BMI, kg·m^2^	29.59 ± 5.25	30.03 ± 6.37	28.74 ± 4.77	27.71 ± 4.55	NS
Fat mass, %	38.89 ± 6.25	40.17 ± 7.20	37.73 ± 5.28	37.65 ± 3.93	NS

*BMI: body mass index; AitH: Aerobic interval training Hypoxia group; AitN: Aerobic interval training Normoxia group; SitH: Sprint interval training Hypoxia group; SitN: Sprint interval training Normoxia group. P-values of analysis of variance to compare differences between groups at baseline*.

a *Indicates differences with respect to AitH (post hoc t-test with Bonferroni correction)*.

b *Indicates differences with respect to SitH (post hoc t-test with Bonferroni correction).Values in bold are significant*.

Oxygen saturation during the training session was lower (*p* < 0.01) and RPE was higher (*p* > 0.05) for the hypoxia groups. HR and training power during training sessions were not significantly different between groups (Table 2).

**Table 2:** Average training control variables during the program.

	**AitN** **(*n* = 13)**	**AitH** **(*n* = 13)**	**SitN** **(*n* = 15)**	**SitH** **(*n* = 18)**	***P*-value**
	**Mean ± SD**	**Mean ± SD**	**Mean ± SD**	**Mean ± SD**	
Pmax, watts	88.59 ± 18.38	89.76 ± 18.47	89.30 ± 27.73	86.31 ± 19.57	NS
HRave, bmp	131.6 ± 11.99	138.90 ± 10.49	133.76 ± 12.64	139.86 ± 11.54	NS
HR max, %	81.91 ± 12.97	86.25 ± 6.32	86.77 ± 6.68	85.46 ± 7.53	NS
SO_2_, %	95.20 ± 3.19^b^	88.86 ± 2.61^a^	95.14 ± 2.83^d^	88.66 ± 1.75^c^	***p* < 0.001**
RPE, Borg scale	4.29 ± 1.60^b^	5.42 ± 1.32^a^	4.04 ± 1.26	4.83 ± 1.31	***p* < 0.01**

*Pmax: maximal power; HRave; average heart rate during total training session; HR max: maximal heart rate during main part training session; SO_2_: oxygen saturation during training session; RPE: Rated Perceived Exertion; EE: energy expenditure; AitH: Aerobic interval training Hypoxia group; AitN: Aerobic interval training Normoxia group; SitH: Sprint interval training Hypoxia group; SitN: Sprint interval training Normoxia group. P-values of analysis of variance to compare differences between groups at baseline*.

a *Indicates differences with respect to AitN (post-hoc t-test with Bonferroni correction)*.

b *Indicates differences with respect to AitH (post-hoc t-test with Bonferroni correction)*.

c *Indicates differences with respect to SitN (post-hoc t-test with Bonferroni correction)*.

d *Indicates differences with respect to SitH (post-hoc t-test with Bonferroni correction)*.

*Values in bold are significant*.

Energy intake and estimated physical activity level prior to and after the intervention are presented in Table 3. No significant difference was noted before and after the intervention.

**Table 3:** Energy intake and physical activity prior to the intervention (Pre) and after the intervention (Post).

		**AitN** **(*n* = 13)**	**AitH** **(*n* = 13)**	**SitN** **(*n* = 15)**	**SitH** **(*n* = 18)**
		**Mean ± SD**	**Mean ± SD**	**Mean ± SD**	**Mean ± SD**
Energy intake, kcal·day^−1^	Pre	1488.24 ± 539.37	1955.55 ± 1647.71	1377.66 ± 612.67	1966.20 ± 1141.43
	Post	1456.69 ± 389.07	1608.07 ± 962.85	1311.56 ± 476.82	2283.50 ± 1496.75
Physical activity, mets·day	Pre	6081.23 ± 1452.77	6158.72 ± 598.31	6722.62 ± 1355.74	6711.84 ± 1543.01
	Post	6029.96 ± 1457.48	6404.20 ± 1179.68	6985.14 ± 605.62	6779.83 ± 2642.24

*AitH: Aerobic interval training Hypoxia group; AitN: Aerobic interval training Normoxia group; SitH: Sprint interval training Hypoxia group; SitN: Sprint interval training Normoxia group*.

### Body composition parameters

Body mass, BMI, percentage of fat mass, and muscle mass changed across T1, T2, T3, and T4. There were no significant differences in body mass and BMI in the within-subjects or in the within-groups analysis in the normoxia groups. Hypoxia groups showed significant differences in body mass and BMI variations. The AitH group showed significantly increased body weight (*p* < 0.001) and BMI (*p* < 0.001) from T1 to T3 and T4. The SitH group showed slight reductions in body weight and BMI in the same time comparison.

**Figure 3:**
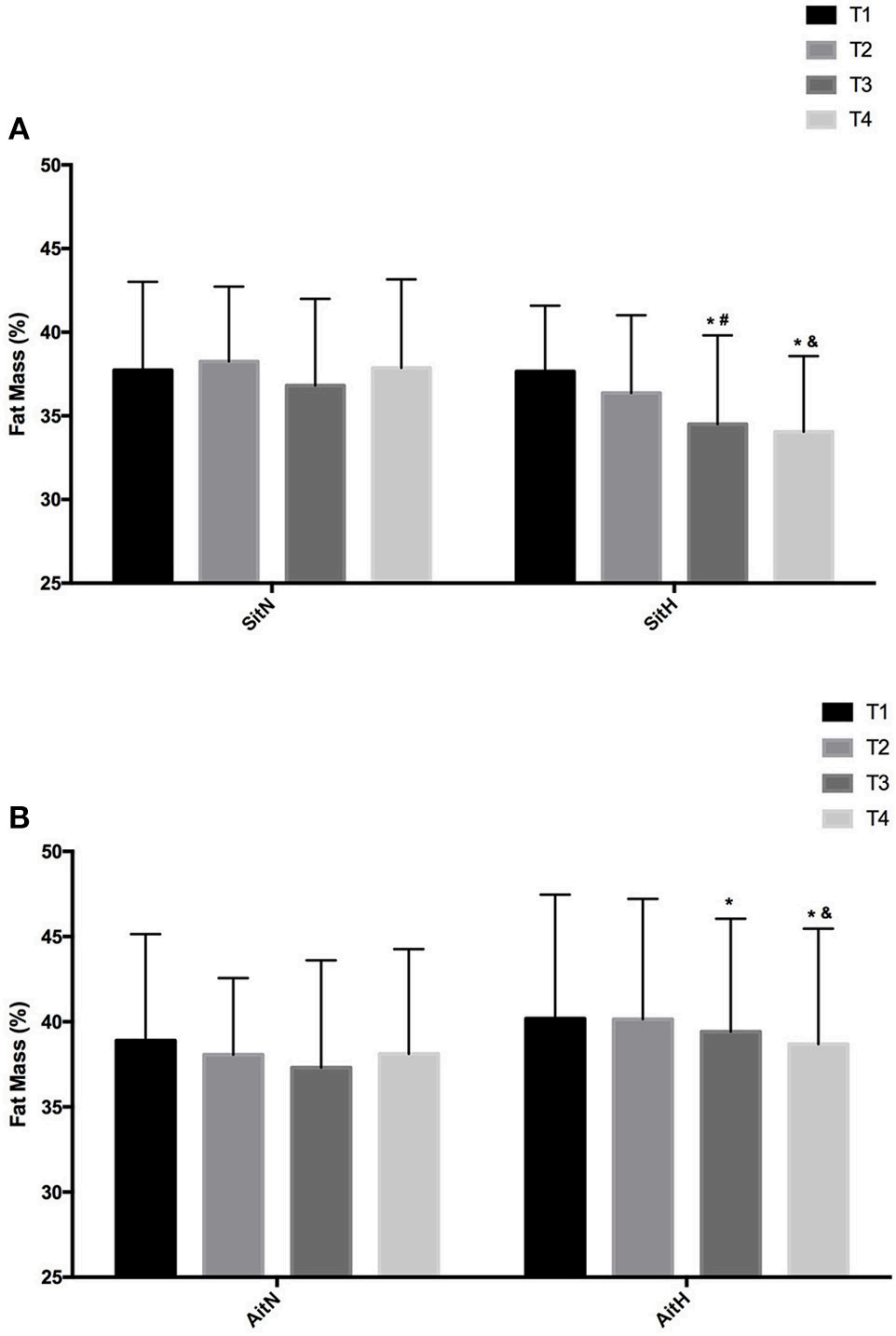
Training effect in fat mass measured at baseline, before 18 sessions (T2) and a week (T3) and 4 weeks (T4) after intervention in each group [**(A)** SitN: sprint interval training normoxia; SitH: sprint interval training hypoxia; **(B)** AitN: aerobic interval training normoxia; AitH: aerobic interval training hypoxia]. ^*^Significant difference respect to baseline (*P* < 0.05). ^#^Significant difference between group in T3 (*P* < 0.05); ^&^Significant difference between group in T4 (*P* < 0.05).

**Figure 4:**
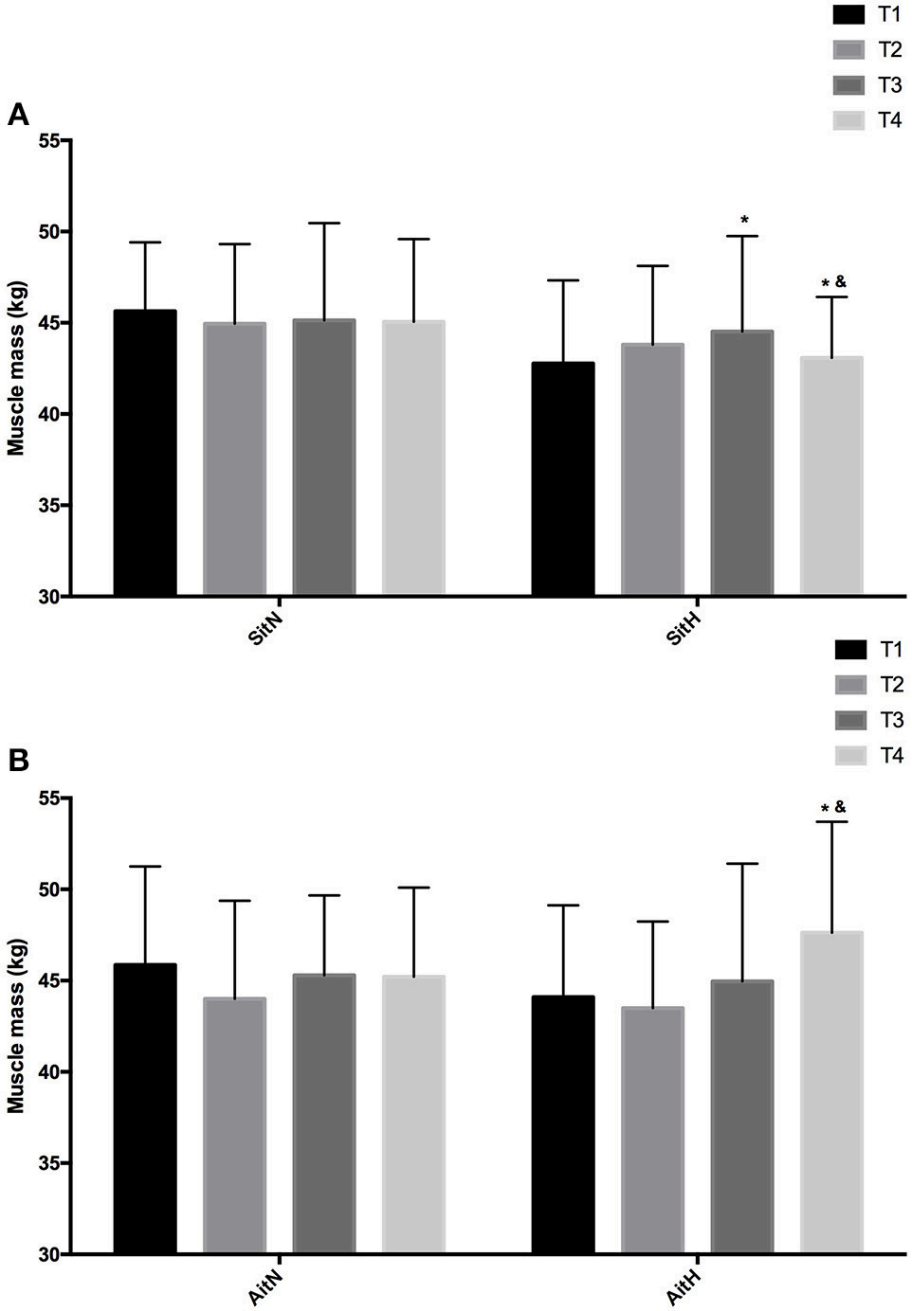
Training effect in muscle mass measured at baseline, before 18 sessions (T2) and a week (T3) and 4 weeks (T4) after intervention in each group [**(A)** SitN: sprint interval training normoxia; SitH: sprint interval training hypoxia; **(B)** AitN: aerobic interval training normoxia; AitH: aerobic interval training hypoxia). ^*^Significant difference respect to baseline (*P* < 0.05). ^&^Significant difference between group in T4 (*P* < 0.05).

There were statistically significant differences between groups in the percentage of fat mass (Figure 3) and muscle mass (Figure 4). The reduction of fat mass in the SitH group was statistically significant when compared with the SitN group from T1 to T3 (*p* < 0.05) and from T1 to T4 (*p* < 0.05). Fat mass in the AitH group showed a statistically significant decrease (*p* < 0.01) that was higher than the AitN group from T1 to T4. There were statistically significant differences in the percentage of change in muscle mass between hypoxia and normoxia groups from T1 to T4. All training groups showed a reduction in the percentage of fat mass, which was statistically significant in the hypoxia groups (Figure 2, *p* < 0.05). Muscle mass presented a statistically significant increase in the hypoxia groups (Figure 3, *p* < 0.05), especially at T4.

### Basal energy expenditure and substrate utilisation

Effects of different trainings on RQ, BMR, energy and fat oxidation, and energy and carbohydrate oxidation are presented in Table 4. There were significant differences in BMR between the AitN and AitH groups when T1 was compared with T2 and T4 (*p* < 0.05). Basal metabolic rate tended to decrease in normoxia groups, especially in the AitN group from T1 to T4, which showed a decrease of 13.17%, but these changes were not statistically significant. There was a statistically significant increase (*p* < 0.05) in BMR in the AitH group at T4 by 8.03%. The statistical analysis revealed that RQ tended to increase in both normoxia groups and decrease in both hypoxia groups after completion of the training protocols, although there were no significant changes within-subjects or between groups.

**Table 4:** Changes on resting energy expenditure and substrate utilization over 12-weeks intervention period.

		**T1** **(A)**	**T2** **(B)**	**Δ** **(A-B)**	**T3** **(C)**	**Δ** **(A-C)**	**T4** **(D)**	**Δ** **(A-D)**	**ANOVA, *p*-values**	
		**Mean ± SD**	**Mean ± SD**	**%**	**Mean ± SD**	**%**	**Mean ± SD**	**%**	**Time**	**Time x group**
RQ	AitN	0.98 ± 0.09	0.93 ± 0.10	−5.10	0.99 ± 0.06	1.02	0.99 ± 0.09	1.02	NS	NS
	AitH	0.97 ± 0.09	0.97 ± 0.08	0.00	0.95 ± 0.06	−2.06	0.95 ± 0.07	−2.06	NS	
	SitN	0.97 ± 0.08	0.95 ± 0.09	−2.06	1.01 ± 0.07	4.12	0.98 ± 0.08	1.03	NS	NS
	SitH	1.01 ± 0.09	1.02 ± 0.07	0.99	0.97 ± 0.08	−3.96	0.96 ± 0.09	−4.95	NS	
BMR, kcal/day	AitN	1676 ± 252	1589 ± 278α	−5.19	1677 ± 293	0.06	1455 ± 320α	−13.17	NS	***P* < 0.01**
	AitH	1588 ± 320	1690 ± 305α	6.46	1530 ± 347	−3.63	1715 ± 404α	8.03	NS	
	SitN	1692 ± 307	1593 ± 240	−5.82	1702 ± 243	0.58	1534 ± 184	−9.33	NS	
	SitH	1761 ± 288	1699 ± 390	−3.54	1707 ± 343	−3.09	1710 ± 362	−2.94	NS	
Energy Fat (kcal/min)	AitN	0.46 ± 0.26	0.59 ± 0.26	28.26	0.33 ± 0.22^*^α	−28.26	0.29 ± 0.12α	−36.96	**0.000**	***p* < 0.001**
	AitH	0.43 ± 0.18	0.43 ± 0.26	0.00	0.46 ± 0.19α	6.98	0.57 ± 0.25α	32.56	NS	
	SitN	0.48 ± 0.25	0.39 ± 0.47	−18.75	0.29 ± 0.19	−39.58	0.38 ± 0.19	−20.83	NS	NS
	SitH	0.37 ± 0.19	0.38 ± 0.19	2.70	0.45 ± 0.24	21.62	0.55 ± 0.25	48.65	NS	
FAToxi (g/min)	AitN	0.052 ± 0.03	0.064 ± 0.03α	40.30	0.036 ± 0.02^*^α	−30.75	0.031 ± 0.01α	−21.72	**0.000**	***p* < 0.001**
	AitH	0.047 ± 0.02	0.048 ± 0.03α	2.13	0.051 ± 0.02α	8.51	0.063 ± 0.03α	34.04	NS	
	SitN	0.054 ± 0.03	0.043 ± 0.05	−20.37	0.033 ± 0.02	−38.89	0.043 ± 0.02	−20.37	NS	NS
	SitH	0.040 ± 0.02	0.042 ± 0.02	5.00	0.050 ± 0.03	25.00	0.062 ± 0.03	55.00	NS	
Energy CHO (kcal/min)	AitN	0.73 ± 0.25	0.51 ± 0.26α	−30.14	0.87 ± 0.17^*^	19.18	0.76 ± 0.32	4.11	**0.000**	***p* < 0.001**
	AitH	0.70 ± 0.22	0.79 ± 0.34α	12.86	0.62 ± 0.24	−11.43	0.63 ± 0.30	−10.00	NS	
	SitN	0.71 ± 0.28	0.75 ± 0.57	5.63	0.94 ± 0.29	32.39	0.70 ± 0.18	−1.41	NS	NS
	SitH	0.89 ± 0.28	0.83 ± 0.28	−6.74	0.76 ± 0.33	−14.61	0.65 ± 0.32	−26.97	NS	
CHOoxi (g/min)	AitN	0.183 ± 0.06	0.128 ± 0.06	−30.05	0.219 ± 0.04^*^	19.67	0.188 ± 0.08	2.73	**0.000**	***p* < 0.001**
	AitH	0.174 ± 0.05	0.191 ± 0.09	9.77	0.155 ± 0.06	−10.92	0.157 ± 0.08	−9.77	NS	
	SitN	0.178 ± 0.07	0.185 ± 0.14α	3.93	0.235 ± 0.07	32.02	0.177 ± 0.05α	−0.56	NS	NS
	SitH	0.223 ± 0.07	0.207 ± 0.07α	−7.17	0.190 ± 0.08	−14.80	0.161 ± 0.08α	−27.80	NS	

*RQ: respiratory quotient; BMR: basal metabolic rate; FAToxi: fat oxidation; CHOoxi: carbohydrates oxidation AitH: Aerobic interval training Hypoxia group; AitN: Aerobic interval training Normoxia group; SitH: Sprint interval training Hypoxia group; SitN: Sprint interval training Normoxia group*.

*Δ, T2/T3/T4 minus T1 absolute change*.

* *Indicates differences with respect to baseline (post hoc t-test with Bonferroni correction)*.

*α: Indicates differences AitN respect to AitH*.

*Values in bold are statistically significant*.

**Figure 5:**
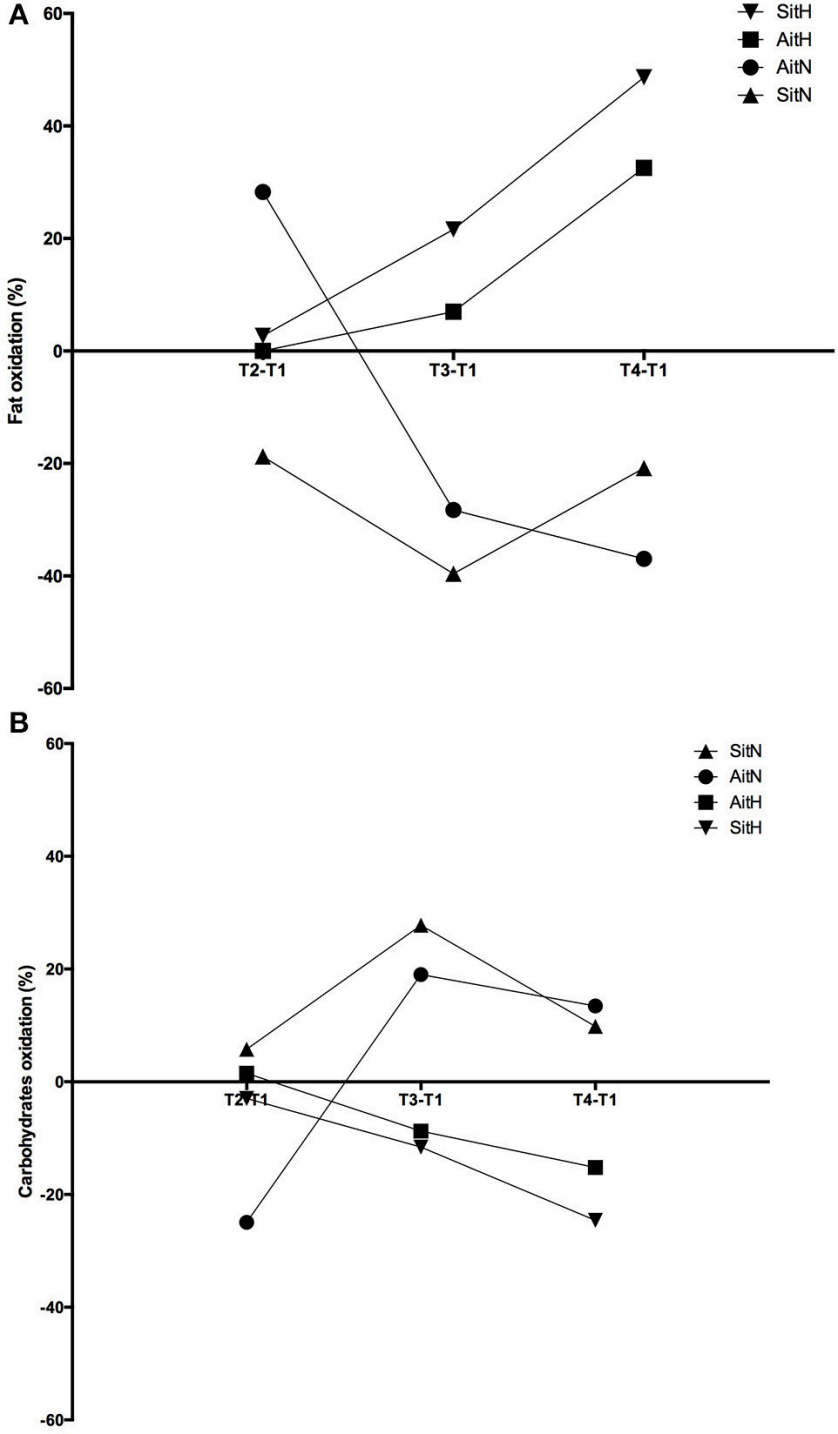
Training effect in percentage of change of fat **(A)** and carbohydrates oxidation **(B)** between baseline and before 18 sessions (T2), baseline and before week (T3) and baseline and before 4 weeks (T4) after intervention in each group (AitN: aerobic interval training normoxia; AitH: aerobic interval training hypoxia; SitN: sprint interval training normoxia; SitH: sprint interval training hypoxia).

Significant differences were found in the percentage of change of fat oxidation and energy from fat (*p* < 0.001) and in the oxidation of carbohydrates and energy from carbohydrates (*p* < 0.001) between the AitH and AitN groups. While fat oxidation tended to increase and oxidation of carbohydrates tended to decrease in both hypoxia groups, this tendency was reversed in normoxia groups (Figure 5). However, the changes were statistically significant (*p* < 0.001) only in the AitN group, where fat oxidation decreased by 36.96% and oxidation of carbohydrates increased by 13.43% after training. Consequently, energy from fat and carbohydrates changed proportionally (*p* < 0.001 in the AitN group).

## Discussion

To the best of our knowledge, this paper represents the first four-group randomised controlled work investigating the effects of simulated hypoxia combined with HIIT on human body composition. The main findings confirm the hypothesis that a 12-week program of HIIT in hypoxia reduces fat mass and increases muscle mass to a greater extent in overweight/obese women than exercising in normoxia. The greatest effects were apparent immediately after completion of the training program. The increase in fat oxidation and the decrease in carbohydrate oxidation in the hypoxia groups could explain these results.

White adipose tissue is known to present a hypoxic state in obese subjects. Although this chronic state may favour other diseases, intermittent hypoxia has been suggested as a treatment option for being overweight and obesity (Heinonen et al., 2016). Reductions in body weight observed at high altitudes (also termed “altitude anorexia”) seem to be a consequence of blunted appetite resulting in decreased energy intake (Benso et al., 2007). Hypoxia alters the function of the nervous system and hormonal levels, which lead to a disturbance of the energy balance explained by a reduction in nutritional and intestinal energy uptake, and increased energy expenditure (Kayser and Verges, 2013). During periods of hypoxia, the activation of HIF could cause a shift in metabolism away from the glycolytic pathway (Wheaton and Chandel, 2011). These 2 sentences are not really understandable to me; away from glycolysis of favouring glycolysis? Please check carefully.

Since glycolysis only produces two adenosine triphosphate (ATP) molecules for every mole of glucose, upregulation of HIF leads to a greater dependency of glucose uptake to generate adequate amounts of ATP (Wheaton and Chandel, 2011). However, this change in metabolism is inefficient since only two ATPs are formed for each mole of glucose metabolised and six ATPs are used for every two molecules of lactate converted to glucose. This energy wasting may play a role in the increase in BMR, which occurs at high altitude (Palmer and Clegg, 2014).

Although studies investigating training under hypoxic conditions on clinical pathologies are rare (Wiesner et al., 2010), some studies have been conducted to test the effects of activities in hypoxia as a treatment for weight loss and improvements in body composition in overweight/obese subjects (Netzer et al., 2008; Wiesner et al., 2010; Gatterer et al., 2015; Kong et al., 2016). In these studies, hypoxic exposure (FiO_2_ = 12–15%) was combined with cardiovascular exercise programs (running, cycling, or stepping) of moderate intensity (55–65% VO_2_ max/60–70% HR max; 60–90 min). Kong et al. (2016) added strength training (40–50% of 1 RM, 3 sets of 15 repetitions, interspersed with 2–3 min rest periods). Overall, published findings, at present, show changes in responses of body weight and BMI following moderate-intensity and cardio-based exercise programs in hypoxia (Netzer et al., 2008; Wiesner et al., 2010). In addition, a higher decrease in fat mass has been shown in obese patients who exercised under hypoxic conditions, but lean body mass did not change. However, the effectiveness of such low-intensity interventions remains questionable, mainly over a longer period. The results of Gatterer et al. (2015) did not demonstrate improvements in body weight between hypoxic and normoxic exposure over a period of 8 months. A reduced workload of participants carrying out moderate-intensity continuous exercise in hypoxia and adaptations to the same stimulus could explain the findings of the present study (Wiesner et al., 2010; Morishima et al., 2014). In contrast, the present research found additive effects of hypoxia combined with higher exercise intensity. A greater decrease in fat mass and an increase in muscle mass were shown in the hypoxia group compared with the normoxia group even 4 weeks after finishing the training program. Briefly, the present findings indicated a greater long-term effect of combined HIIT with normobaric hypoxia exposure compared with the same protocol in normoxia. The metabolic efficient characteristics of the high-intensity intermittent protocols had a marked advantage in the development of a habitual strategic exercise for combatting obesity (Zhang et al., 2017), which also seems to be more suitable in the hustle and bustle of modern life (Gibala et al., 2012). The impact of HIIT in normoxia on body composition is controversial. Some evidence suggests that it could be an effective strategy for the management of body fat levels in overweight/obese adults (Heydari et al., 2012; Sijie et al., 2012; Gillen et al., 2013; Fisher et al., 2015; Lanzi et al., 2015; Martins et al., 2016; Zhang et al., 2017), but others have not found differences (Whyte et al., 2010; Keating et al., 2014; Smith-Ryan et al., 2015, 2016; Kong et al., 2016). Based on a recent systematic review (Batacan et al., 2017), long-term HIIT protocols reduce the fat percentage in overweight/obese populations. Therefore, at least three times a week for more than 12 weeks of HIIT in normoxic conditions should be performed as part of an exercise program to promote significant fat reductions in overweight/obese populations. In the present study, all training groups showed reductions in percentage of fat mass, but these were only statistically significant in the hypoxia groups, with larger differences in the hypoxia groups compared with the normoxia groups.

The beneficial effects of combined intermittent hypoxia with HIIT on greater reduction of body fat could be attributed to the increased post-exercise lipid oxidation (Kendzerska et al., 2016). During exercise in hypoxia, a greater dependence on carbohydrate substrates (Kelly and Basset, 2017) and a shift away from fat oxidation (Horscroft and Murray, 2014) are commonly observed. This oxygen debt (due to a contribution from non-oxidative mechanisms) will be repaid during recovery from exercise. Thus, depleting glycogen during active hypoxia exposure has been shown to increase resting FAToxi after an exercise period. In this moment, muscle glycogen replenishment is vital and thus, plasma and intramuscular triglycerides are likely to be essential fuel sources for oxidative energy production (Kimber et al., 2003). A recent study (Kelly and Basset, 2017) has shown that substrate oxidation during the post-exercise recovery period has been altered, displaying an increased contribution from FAToxi and a suppressed CHOoxi. Similarly, in the present study, fat oxidation tended to increase and oxidation of carbohydrates tended to decrease in both hypoxia groups. In the normoxia groups, there was an inverse tendency, with a statistically significant difference between the AitH and AitN groups sin substrate oxidation.

Sarcopenia is common in obese patients (Gallagher and DeLegge, 2011). In the present study, vigorous exercise showed a significant improvement in muscle mass, which may have caused the body weight gain, in overweight/obese sedentary women in both hypoxia groups. Previous evidence has shown that chronic hypoxia generally leads to negative regulation of protein metabolism and a loss of muscle mass. Conversely, acute hypoxia seems to exert a positive effect on protein balance in humans when combined with exercise (Nishimura et al., 2010). An acidic environment produced by the accumulation of metabolic waste products (such as lactate) due to anaerobic metabolism, could stimulate the production of growth hormone (Takarada et al., 2000). In this sense, although endurance exercise is generally recognised to cause significant adaptations in skeletal muscle capillarisation (Kon et al., 2015), high-intensity interval training under hypoxic conditions may elicit synergistic effects, producing structural muscle adaptations by stimulating glucose-dependent metabolic pathways and consequently an acidic environment (Vogt et al., 2001).

To the best of our knowledge, there are no studies suggesting why hypoxia may have a beneficial effect on muscle mass. Further investigations are needed to determine the molecular mechanisms that regulate skeletal muscle mass during/after intermittent hypoxia combined with endurance exercise.

In addition, exercise strategies such as those carried out in this study may be especially beneficial for individuals who are unable to maintain adequate workloads for compliance with exercise prescriptions. Due to disproportionately heavier limbs, obese patients need to increase the mechanical demand during exercise to enhance exercise intensity and to meet the current physical activity guidelines (Girard et al., 2017). This increased mechanical demand during exercise in obese populations may be deleterious and can cause pain in lower limb joints (even leading to musculoskeletal pathologies) (Girard et al., 2017). In this sense, the implementation of high intensity exercise could lead to difficulties in maintaining the prescribed work in untrained obese populations. This is where a hypoxic stimulus could be especially beneficial, as it would increase the metabolic stress (relative workload) without intensifying the mechanical stress. Exposure to hypoxia at heights exceeding 2,000 m cause reductions in SpO_2_. To maintain delivery of O_2_ during hypoxic exercise, breathing frequency (Bf), VE, HR, and CHOoxi are all elevated above similar workloads performed at sea level (Peronnet et al., 2006; Mazzeo, 2008). Therefore, hypoxic conditions during endurance training programs could be considered as an additional metabolic stress that the body must overcome to achieve an adequate energy supply (Kelly and Basset, 2017). Briefly, including a hypoxic stimulus during endurance training protocols may be beneficial to overweight/obese patients who have problems achieving the workloads required to obtain cardiovascular and metabolic health benefits (Kelly and Basset, 2017). With respect to the type of exercise, the level of impact produced by the activity could influence joints, such as the knee (Arokoski et al., 2000). Therefore, low impact activities, such as walking or cycling, have been recognised as applying lower loads on the tibiofemoral surfaces of the knee joint, thereby decreasing the risk of injury (Vannini et al., 2016). In addition, repeated-sprint ability (RSA) cycling was found to induce larger decrements in muscle contractile properties in the knee joint as well as higher levels of low-frequency fatigue compared with running (Rampinini et al., 2016). For this reason, the use of these types of exercises is recommended in this population.

Our study has some limitations. Information on food intake and physical activity patterns were only registered twice during the intervention protocol. Even though participants were informed not to change their normal physical activity and eating habits and non-significant differences were found between records, some favourable behavioural alterations might have occurred outside the study procedure. However, maintaining the subjects on their normal food habits seems to be the most logical choice, and their effect should be low and not influence current conclusions. Based on previous studies, protocols with 30-s high-intensity intervals may be more enjoyable than longer protocols (Martinez et al., 2015). One important aspect may be related to the individual's ability or competency to successfully complete the training. In fact, the positive feedback to complete a task increases enjoyment (Hu et al., 2007) and, in this way, the adherence to exercise. On the other hand, the mechanical load in hypoxic conditions may be reduced and thus, the lower physical stress may actually decrease negative feelings and increase enjoyment for the exercise over time (Girard et al., 2017). Therefore, the larger variation of energy intake and physical activity in SitH group could at least partly be due more distinct differences in the adherence to high-intensity exercise in hypoxia. Anyway, despite the large variation of both variables, especially in SitH group, no significant differences were observed in any group. Even though the dropout rate of study (28.05%) was lower than previous studies (Miller et al., 2014; Gatterer et al., 2015), the loss of sample might have led to a selection bias toward those already more motivated and prepared to carry on and those who could have gained more from the program because of a higher rate of obesity. However, we do not believe that this alters the outcome of the research.

We conclude that HIIT under normobaric intermittent hypoxia for 12 weeks is promising for reducing body fat content with a concomitant increase in muscle mass. The reduction in the risk for orthopaedic injury and time and metabolic efficiency, which was enhanced by hypoxia in the SIT and AIT-HIIT programs, may be particularly feasible for overweight/obese patients for whom exercise capacity is limited by orthopaedic conditions. However, further studies are needed to validate these findings and to determine the most appropriate dose of hypoxia and high-intensity exercise for this population.

## Author contributions

AC-C: Designed the research study, conducted the experiments, and wrote the manuscript; MC-C: Acquired and analyse the data, and wrote the manuscript; MB: Analysed the data, and wrote the manuscript; IM-G: Conducted the experiments, and acquired the data; RT: Designed the research study, and acquired the data; JB-S: Designed the research study, acquired the data, and wrote the manuscript; GO: Designed the research study, analysed the data, and wrote the final version of the manuscript.

### Conflict of interest statement

The authors declare that the research was conducted in the absence of any commercial or financial relationships that could be construed as a potential conflict of interest.

## References

[B1] ACSM (2014). ACSM's Guidelines for Exercise Testing and Prescription. 8th Edn. Baltimore: Lippincott Williams & Wilkins.

[B2] Arokoski J. P. Jurvelin J. S. Väätäinen U. Helminen H. J. (2000). Normal and pathological adaptations of articular cartilage to joint loading. Scand. J. Med. Sci. Sports 10, 186–198. 10.1034/j.1600-0838.2000.010004186.x 10898262

[B3] Batacan R. B. Jr Duncan M. J. Dalbo V. J. Tucker P. S. Fenning A. S. (2017). Effects of high-intensity interval training on cardiometabolic health: a systematic review and meta-analysis of intervention studies. Br. J. Sports Med. 51, 494–503. 10.1136/bjsports-2015-095841 27797726

[B4] Benso A. Broglio F. Aimaretti G. Lucatello B. Lanfranco F. Ghigo E.. (2007). Endocrine and metabolic responses to extreme altitude and physical exercise in climbers. Eur. J. Endocrinol. 157, 733–740. 10.1530/EJE-07-0355 18057380

[B5] Borg G. A. (1982). Psychophysical bases of perceived exertion. Med. Sci. Sports Exerc. 14, 377–381. 10.1249/00005768-198205000-00012 7154893

[B6] Bouchard C. Depres J. P. Tremblay A. (1993). Exercise and obesity. Obes. Res. 1, 133–147. 10.1002/j.1550-8528.1993.tb00603.x 16350569

[B7] Brandou F. Savy-Pacaux A. M. Marie J. Bauloz M. Maret-Fleuret I. Borrocoso S.. (2005). Impact of high- and low-intensity targeted exercise training on the type of substrate utilization in obese boys submitted to a hypocaloric diet. Diabetes Metab. 31(4 Pt 1), 327–335. 10.1016/S1262-3636(07)70201-X 16369194

[B8] Camacho-Cardenosa M. Camacho-Cardenosa A. Martínez Guardado I. Marcos-Serrano M. Timon R. Olcina G. (2017). A new dose of maximal-intensity interval training in hypoxia to improve body composition and hemoglobin and hematocrit levels: a pilot study. J. Sports Med. Phys. Fitness 57, 60–69. 10.23736/S0022-4707.16.06549-X 27441916

[B9] Carter S. L. Rennie C. Tarnopolsky M. A. (2001). Substrate utilization during endurance exercise in men and women after endurance training. Am. J. Physiol. Endocrinol. Metab. 280, E898–E907. 10.1152/ajpendo.2001.280.6.E898 11350771

[B10] Craig C. L. Marshall A. L. Sjostrom M. Bauman A. E. Booth M. L. Ainsworth B. E.. (2003). International physical activity questionnaire: 12-country reliability and validity. Med. Sci. Sports Exerc. 35, 1381–1395. 10.1249/01.MSS.0000078924.61453.FB 12900694

[B11] Fisher G. Brown A. W. Bohan Brown M. M. Alcorn A. Noles C. Winwood L.. (2015). High intensity interval- vs. moderate intensity- training for improving cardiometabolic health in overweight or obese males: a randomized controlled trial. PLoS ONE 10:e0138853. 10.1371/journal.pone.0138853 26489022 PMC4619258

[B12] Gallagher D. DeLegge M. (2011). Body composition (sarcopenia) in obese patients: implications for care in the intensive care unit. JPEN J. Parenter. Enteral. Nutr. 35(5 Suppl.), 21S−28S. 10.1177/0148607111413773 21807929 PMC3306216

[B13] Gatterer H. Haacke S. Burtscher M. Faulhaber M. Melmer A. Ebenbichler C.. (2015). Normobaric intermittent hypoxia over 8 months does not reduce body weight and metabolic risk factors–a randomized, single blind, placebo-controlled study in normobaric hypoxia and normobaric sham hypoxia. Obes. Facts 8, 200–209. 10.1159/000431157 26008855 PMC5644878

[B14] Gibala M. J. Little J. P. Macdonald M. J. Hawley J. A. (2012). Physiological adaptations to low-volume, high-intensity interval training in health and disease. J. Physiol. 590, 1077–1084. 10.1113/jphysiol.2011.224725 22289907 PMC3381816

[B15] Gillen J. B. Percival M. E. Ludzki A. Tarnopolsky M. A. Gibala M. J. (2013). Interval training in the fed or fasted state improves body composition and muscle oxidative capacity in overweight women. Obesity (Silver. Spring). 21, 2249–2255. 10.1002/oby.20379 23723099

[B16] Girard O. Malatesta D. Millet G. P. (2017). Walking in hypoxia: an efficient treatment to lessen mechanical constraints and improve health in obese individuals? Front. Physiol. 8:73. 10.3389/fphys.2017.00073 28232806 PMC5298970

[B17] Haugen H. A. Chan L. N. Li F. (2007). Indirect calorimetry: a practical guide for clinicians. Nutr. Clin. Pract. 22, 377–388. 10.1177/0115426507022004377 17644692

[B18] Heinonen I. H. Boushel R. Kalliokoski K. K. (2016). The circulatory and metabolic responses to hypoxia in humans - with special reference to adipose tissue physiology and obesity. Front. Endocrinol. (Lausanne). 7:116. 10.3389/fendo.2016.00116 27621722 PMC5002918

[B19] Heydari M. Freund J. Boutcher S. H. (2012). The effect of high-intensity intermittent exercise on body composition of overweight young males. J. Obes. 2012:480467. 10.1155/2012/480467 22720138 PMC3375095

[B20] Hobbins L. G. Hunter S. Gaoua N. Girard O. (2017). Normobaric hypoxic conditioning to maximise weight-loss and ameliorate cardio-metabolic health in obese populations: a systematic review. Am. J. Physiol. Regul. Integr. Comp. Physiol. 313, R251–R264. 10.1152/ajpregu.00160.2017 28679682

[B21] Horner N. K. Lampe J. W. Patterson R. E. Neuhouser M. L. Beresford S. A. Prentice R. L. (2001). Indirect calorimetry protocol development for measuring resting metabolic rate as a component of total energy expenditure in free-living postmenopausal women. J. Nutr. 131, 2215–2218. 10.1093/jn/131.8.2215 11481420

[B22] Horscroft J. A. Murray A. J. (2014). Skeletal muscle energy metabolism in environmental hypoxia: climbing towards consensus. Extrem. Physiol. Med. 3:19. 10.1186/2046-7648-3-19 25473486 PMC4253994

[B23] Hu L. Motl R. W. McAuley E. Konopack J. F. (2007). Effects of self-efficacy on physical activity enjoyment in college-aged women. Int. J. Behav. Med. 14, 92–96. 10.1007/BF03004174 17926437

[B24] Jakicic J. M. Clark K. Coleman E. Donnelly J. E. Foreyt J. Melanson E.. (2001). American college of sports medicine position stand. appropriate intervention strategies for weight loss and prevention of weight regain for adults. Med. Sci. Sports Exerc. 33, 2145–2156. 10.1097/00005768-200112000-00026 11740312

[B25] Kanter R. Caballero B. (2012). Global gender disparities in obesity: a review. Adv. Nutr. 3, 491–498. 10.3945/an.112.002063 22797984 PMC3649717

[B26] Kayser B. Verges S. (2013). Hypoxia, energy balance and obesity: from pathophysiological mechanisms to new treatment strategies. Obes. Rev. 14, 579–592. 10.1111/obr.12034 23551535

[B27] Keating S. E. Machan E. A. O'Connor H. T. Gerofi J. A. Sainsbury A. Caterson I. D. (2014). Continuous exercise but not high intensity interval training improves fat distribution in overweight adults. J. Obes. 2014:834865 10.1155/2014/834865 24669314 PMC3942093

[B28] Kelly L. P. Basset F. A. (2017). Acute normobaric hypoxia increases post-exercise lipid oxidation in healthy males. Front. Physiol. 8:293. 10.3389/fphys.2017.00293 28567018 PMC5434119

[B29] Kendzerska T. Leung R. S. Gershon A. S. Tomlinson G. Ayas N. (2016). The interaction of obesity and nocturnal hypoxemia on cardiovascular consequences in adults with suspected obstructive sleep apnea. a historical observational study. Ann. Am. Thorac. Soc. 13, 2234–2241. 10.1513/AnnalsATS.201604-263OC 27690206

[B30] Kessler H. S. Sisson S. B. Short K. R. (2012). The potential for high-intensity interval training to reduce cardiometabolic disease risk. Sports Med. 42, 489–509. 10.2165/11630910-000000000-00000 22587821

[B31] Kimber N. E. Heigenhauser G. J. Spriet L. L. Dyck D. J. (2003). Skeletal muscle fat and carbohydrate metabolism during recovery from glycogen-depleting exercise in humans. J. Physiol. 548(Pt 3), 919–927. 10.1113/jphysiol.2002.031179 12651914 PMC2342904

[B32] Kon M. Ohiwa N. Honda A. Matsubayashi T. Ikeda T. Akimoto T.. (2015). Effects of systemic hypoxia on human muscular adaptations to resistance exercise training. Physiol. Rep. 3:e12267. 10.14814/phy2.12267 25602020 PMC4387762

[B33] Kong Z. Sun S. Liu M. Shi Q. (2016). Short-term high-intensity interval training on body composition and blood glucose in overweight and obese young women. J. Diabetes Res. 2016:4073618. 10.1155/2016/4073618 27774458 PMC5059579

[B34] Lanzi S. Codecasa F. Cornacchia M. Maestrini S. Capodaglio P. Brunani A.. (2015). Short-term HIIT and Fat max training increase aerobic and metabolic fitness in men with class II and III obesity. Obesity (Silver. Spring). 23, 1987–1994. 10.1002/oby.21206 26335027

[B35] Lanzi S. Codecasa F. Cornacchia M. Maestrini S. Salvadori A. Brunani A.. (2014). Fat oxidation, hormonal and plasma metabolite kinetics during a submaximal incremental test in lean and obese adults. PLoS ONE 9:e88707. 10.1371/journal.pone.0088707 24523934 PMC3921204

[B36] Malnick S. D. Knobler H. (2006). The medical complications of obesity. QJM 99, 565–579. 10.1093/qjmed/hcl085 16916862

[B37] Martinez N. Kilpatrick M. W. Salomon K. Jung M. E. Little J. P. (2015). Affective and Enjoyment responses to high-intensity interval training in overweight-to-obese and insufficiently active adults. J. Sport Exerc. Psychol. 37, 138–149. 10.1123/jsep.2014-0212 25996105

[B38] Martins C. Kazakova I. Ludviksen M. Mehus I. Wisloff U. Kulseng B.. (2016). High-intensity interval training and isocaloric moderate-intensity continuous training result in similar improvements in body composition and fitness in obese individuals. Int. J. Sport Nutr. Exerc. Metab. 26, 197–204. 10.1123/ijsnem.2015-0078 26479856

[B39] Mazzeo R. S. (2008). Physiological responses to exercise at altitude : an update. Sports Med. 38, 1–8. 10.2165/00007256-200838010-00001 18081363

[B40] Miller F. L. O'Connor D. P. Herring M. P. Sailors M. H. Jackson A. S. Dishman R. K.. (2014). Exercise dose, exercise adherence, and associated health outcomes in the TIGER study. Med. Sci. Sports Exerc. 46, 69–75. 10.1249/MSS.0b013e3182a038b9 23793231 PMC3867583

[B41] Millet G. P. Debevec T. Brocherie F. Malatesta D. Girard O. (2016). Therapeutic use of exercising in hypoxia: promises and limitations. Front. Physiol. 7:224. 10.3389/fphys.2016.00224 27375500 PMC4902009

[B42] Morishima T. Kurihara T. Hamaoka T. Goto K. (2014). Whole body, regional fat accumulation, and appetite-related hormonal response after hypoxic training. Clin. Physiol. Funct. Imaging 34, 90–97. 10.1111/cpf.12069 23879294

[B43] Netzer N. C. Chytra R. Küpper T. (2008). Low intense physical exercise in normobaric hypoxia leads to more weight loss in obese people than low intense physical exercise in normobaric Sham Hypoxia. Sleep Breath. 12, 129–134. 10.1007/s11325-007-0149-3 18057976 PMC2276561

[B44] Nishimura A. Sugita M. Kato K. Fukuda A. Sudo A. Uchida A. (2010). Hypoxia increases muscle hypertrophy induced by resistance training. Int. J. Sports Physiol. Perform. 5, 497–508. 10.1123/ijspp.5.4.497 21266734

[B45] Oh C. Jeon B. H. Reid Storm S. N. Jho S. No J. K. (2017). The most effective factors to offset sarcopenia and obesity in the older Korean: physical activity, vitamin D, and protein intake. Nutrition 33, 169–173. 10.1016/j.nut.2016.06.004 27717662

[B46] Palmer B. F. Clegg D. J. (2014). Ascent to altitude as a weight loss method: the good and bad of hypoxia inducible factor activation. Obesity (Silver. Spring). 22, 311–317. 10.1002/oby.20499 23625659 PMC4091035

[B47] Péronnet F. Massicotte D. Folch N. Melin B. Koulmann N. Jimenez C.. (2006). Substrate utilization during prolonged exercise with ingestion of (13)C-glucose in acute hypobaric hypoxia (4,300 m). Eur. J. Appl. Physiol. 97, 527–534. 10.1007/s00421-006-0164-2 16775741

[B48] Rampinini E. Connolly D. R. Ferioli D. La Torre A. Alberti G. Bosio A. (2016). Peripheral neuromuscular fatigue induced by repeated-sprint exercise: cycling vs. running. J. Sports Med. Phys. Fitness 56, 49–59. 25289713

[B49] Sijie T. Hainai Y. Fengying Y. Jianxiong W. (2012). High intensity interval exercise training in overweight young women. J. Sports Med. Phys. Fitness 52, 255–262. 22648463

[B50] Smith-Ryan A. E. Melvin M. N. Wingfield H. L. (2015). High-intensity interval training: modulating interval duration in overweight/obese men. Phys. Sportsmed. 43, 107–113. 10.1080/00913847.2015.1037231 25913937 PMC4427241

[B51] Smith-Ryan A. E. Trexler E. T. Wingfield H. L. Blue M. N. (2016). Effects of high-intensity interval training on cardiometabolic risk factors in overweight/obese women. J. Sports Sci. 34, 2038–2046. 10.1080/02640414.2016.1149609 26934687 PMC5010533

[B52] Takarada Y. Takazawa H. Ishii N. (2000). Applications of vascular occlusion diminish disuse atrophy of knee extensor muscles. Med. Sci. Sports Exerc. 32, 2035–2039. 10.1097/00005768-200012000-00011 11128848

[B53] Trombold J. R. Christmas K. M. Machin D. R. Kim I. Y. Coyle E. F. (2013). Acute high-intensity endurance exercise is more effective than moderate-intensity exercise for attenuation of postprandial triglyceride elevation. J. Appl. Physiol (1985) 114, 792–800. 10.1152/japplphysiol.01028.2012 23372145

[B54] Urdampilleta A. González-Muniesa P. Portillo M. P. Martínez J. A. (2012). Usefulness of combining intermittent hypoxia and physical exercise in the treatment of obesity. J. Physiol. Biochem. 68, 289–304. 10.1007/s13105-011-0115-1 22045452

[B55] Vannini F. Spalding T. Andriolo L. Berruto M. Denti M. Espregueira-Mendes J.. (2016). Sport and early osteoarthritis: the role of sport in aetiology, progression and treatment of knee osteoarthritis. Knee Surg. Sports Traumatol. Arthrosc. 24, 1786–1796. 10.1007/s00167-016-4090-5 27043343

[B56] Vogt M. Puntschart A. Geiser J. Zuleger C. Billeter R. Hoppeler H. (2001). Molecular adaptations in human skeletal muscle to endurance training under simulated hypoxic conditions. J. Appl. Physiol. (1985) 91, 173–182. 10.1152/jappl.2001.91.1.173 11408428

[B57] Walsh K. (2009). Adipokines, myokines and cardiovascular disease. Circ. J. 73, 13–18. 10.1253/circj.CJ-08-0961 19043226

[B58] Wearing S. C. Hennig E. M. Byrne N. M. Steele J. R. Hills A. P. (2006). The biomechanics of restricted movement in adult obesity. Obes. Rev. 7, 13–24. 10.1111/j.1467-789X.2006.00215.x 16436099

[B59] Weir J. B. (1949). New methods for calculating metabolic rate with special reference to protein metabolism. J. Physiol. 109, 1–9. 10.1113/jphysiol.1949.sp004363 15394301 PMC1392602

[B60] Westerterp K. R. Kayser B. (2006). Body mass regulation at altitude. Eur. J. Gastroenterol. Hepatol. 18, 1–3. 10.1097/00042737-200601000-00001 16357611

[B61] Wheaton W. W. Chandel N. S. (2011). Hypoxia. 2. Hypoxia regulates cellular metabolism. Am. J. Physiol. Cell Physiol. 300, C385–C393. 10.1152/ajpcell.00485.2010 21123733 PMC3063979

[B62] WHO P. (2006). Gaining Health. The European Strategy for the Prevention and Control of non-communicable Diseases. World Health Organisation, Regional Office for Europe.

[B63] Whyte L. J. Gill J. M. Cathcart A. J. (2010). Effect of 2 weeks of sprint interval training on health-related outcomes in sedentary overweight/obese men. Metab. Clin. Exp. 59, 1421–1428. 10.1016/j.metabol.2010.01.002 20153487

[B64] Wiesner S. Haufe S. Engeli S. Mutschler H. Haas U. Luft F. C.. (2010). Influences of normobaric hypoxia training on physical fitness and metabolic risk markers in overweight to obese subjects. Obesity (Silver. Spring). 18, 116–120. 10.1038/oby.2009.193 19543214

[B65] Wood K. Olive B. Lavalle K. Thompson H. Greer K. Astorino T. A. (2015). Dissimilar physiological and perceptual responses between sprint interval training and high-intensity interval training J. Strength Cond. Res. 30, 244–250. 10.1519/JSC.0000000000001042 26691413

[B66] Wood R. E. Hills A. P. Hunter G. R. King N. A. Byrne N. M. (2010). Vo2max in overweight and obese adults: do they meet the threshold criteria? Med. Sci. Sports Exerc. 42, 470–477. 10.1249/MSS.0b013e3181b666ad 19952821

[B67] Workman C. Basset F. A. (2012). Post-metabolic response to passive normobaric hypoxic exposure in sedendary overweight males: a pilot study. Nutr. Metab. 9:103. 10.1186/1743-7075-9-103 23157699 PMC3546003

[B68] Zhang H. Tong T. K. Qiu W. Zhang X. Zhou S. Liu Y.. (2017). Comparable effects of high-intensity interval training and prolonged continuous exercise training on abdominal visceral fat reduction in obese young women. J. Diabetes Res. 2017:5071740. 10.1155/2017/5071740 28116314 PMC5237463

